# First Lysine Lactylation Profiling in *Vibrio alginolyticus* and Initial Characterization of VaCobQ as a Candidate Delactylase

**DOI:** 10.3390/microorganisms14040926

**Published:** 2026-04-20

**Authors:** Yujia Zhang, Zhiqing Wei, Jiaxin Fan, Weijie Zhang, Shuai Yang, Jichang Jian, Na Wang, Jianyi Wei, Huanying Pang

**Affiliations:** 1 Fisheries College, Guangdong Ocean University, Zhanjiang 524088, China; 2 Guangdong Provincial Key Laboratory of Aquatic Animal Disease Control and Healthy Culture, Zhanjiang 524025, China; 3 Chinese Academy of Quality and Inspection & Testing, Beijing 100176, China; 4 Guangxi Beihai Nanjiang Aquatic Products Co., Ltd., Beihai 536000, China

**Keywords:** *Vibrio alginolyticus*, lysine lactylation, post-translational modification

## Abstract

*Vibrio alginolyticus* is a common pathogenic bacterium and can cause diseases in aquaculture animals. Lysine lactylation (Kla) is a novel post-translational modification (PTM) that has been confirmed to play critical roles in key biological processes. However, the modification landscape and functions of Kla in *V. alginolyticus* remain unclear. In this study, lactylation modification profiles of the bacterial pathogen *V. alginolyticus* were first systematically characterized; a total of 9308 lactylation sites on 2155 proteins were successfully identified. The lactylation of cAMP receptor protein (CRP) and triosephosphate isomerase (TPI) was verified by Co-immunoprecipitation (Co-IP) and Western blot to validate the lactylome data. Bioinformatic analysis of the Kla sites revealed 32 conserved sequence motifs surrounding the modified residues. Kla proteins were mainly involved in central metabolic pathways, including glycolysis/gluconeogenesis and ribosome biogen regulators were found to contain Kla modification sites. To investigate crosstalk among lysine acylations in *V. alginolyticus*, we integrated Kla, lysine acetylation (Kac), and lysine succinylation (Ksuc) profiles and identified 337 co-modified proteins and 5 co-modified sites. Additionally, phylogenetic analysis of Vibrio alginolyticus CobQ based on protein sequence alignment revealed no homology to the known delactylase CobB. Combined in vitro and in vivo functional validation identified VaCobQ as a candidate delactylase with potential NAD^+^-independent activity. This study establishes a lysine lactylation landscape in *V. alginolyticus*, providing a resource for exploring Kla functions in bacterial metabolism and its possible connections to virulence.

## 1. Introduction

Post-translational modifications (PTMs) are covalent modifications that occur after protein biosynthesis, providing precise regulation of protein function [1,2]. The role of PTMs in cellular processes is significant as they regulate cell signaling, influence protein localization, and maintain cellular function by modifying protein structures and function [3]. With the development of highly sensitive mass spectrometry technology, various metabolites serve as donors for lysine acylation, producing multiple forms of acylation.

Lysine lactylation (Kla) is a recently discovered PTM, which plays a key role in basic biological processes [4]. It is caused by lactate accumulation and involves the covalent attachment of a lactyl group to lysine residues in proteins, modulating protein function, stability, and interactions [5,6]. As a dynamic and reversible regulation process, lysine lactylation is regulated by the opposing activities of “writers,” which add lactyl groups, and “erasers,” which remove them [7]. In eukaryotes, various key regulatory enzymes, such as p300 and HDAC1–3, have been proven to possess lactyl transferase or delactylase activity, and the relevant regulatory networks have been initially clarified [4,8]. In contrast, the identity and functions of regulatory enzymes with Kla in prokaryotes still poorly characterized. Recent studies have revealed the correlation between biofilm formation of *Streptococcus mutans* and dynamic changes in Kla [9] and identified novel Kla regulatory enzymes in *Escherichia coli* [10], suggesting a potential role for Kla in bacterial metabolism. Nevertheless, the global functional landscape of Kla and its regulatory enzymes in prokaryotes has not been explored.

*Vibrio alginolyticus*, a Gram-negative marine bacterium belonging to the genus *Vibrio*, which is widely distributed across oceanic, coastal, and estuarine environments [11]. As a zoonotic bacterium, *V. alginolyticus* is a key factor in causing vibriosis in aquatic species and a critical food safety concern [12,13]. It has the ability to infect a diverse range of cultured marine organisms, encompassing shrimp, fish, shellfish, crustaceans, and reefs [14], causing huge economic losses. In addition, *V. alginolyticus* infections in humans can result in many severe diseases like diarrhea, septicemia, and multiple tissue inflammation [15,16]. Its pathogenicity is complex, which is closely related to the synergy of a variety of pathogenic factors. These pathogenic agents, such as flagella, hemolysins, extracellular enzymes, type III secretion system (T3SS), and others, have been the subject of many reports in recent years [14,17,18,19]. The T3SS of *V. alginolyticus* has become a key virulence component, which is a highly conserved syringe-like transmembrane structure located in the cell envelope of Gram-negative bacterial pathogens [20]. It enables the bacterial effector proteins to be transported directly within the bacteria and host membranes into the cytosol of host cells, triggering host cell death and activating innate and adaptive immune responses [21,22].

With the rapid development of proteomic technologies, the role of PTMs in bacteria is becoming increasingly appreciated. Kla has emerged as a novel PTM, and studies across various organisms have underscored its pivotal roles in metabolic regulation, signal transduction, and stress response mechanisms. Here, we present the first comprehensive lactylome of bacterial pathogen *V. alginolyticus*, 9308 lactylation sites on 2155 proteins were identified through Kla antibody enrichment and LC-MS/MS. Bioinformatics analysis showed that lactylated proteins are enriched in various metabolic pathways. By integrating our lactylome data with known type III secretion system (T3SS) regulators, we identified 8 T3SS-associated proteins bearing Kla sites. Furthermore, we obtained preliminary evidence suggesting that CobQ may function as a delactylase in *V. alginolyticus*, demonstrating that recombinant CobQ reduces lysine lactylation levels on lactylated proteins in vitro and that deletion of *cobQ* results in elevated global lysine lactylation levels in vivo, suggesting a potential regulatory role in lactylation dynamics.

This study provides the first comprehensive lysine lactylation landscape in *V. alginolyticus* and identifies VaCobQ as a candidate delactylase, offering a foundation for future investigations into the dynamic regulation of lactylation in prokaryotes and its potential link to pathogenicity.

## 2. Materials and Methods

### 2.1. Bacterial Strains and Protein Extraction

*Vibrio alginolyticus* strain HY9901 was isolated from *Lutjanus erythopterus*, HY9901 Δ*cobQ* was constructed based on prior work. The strain was first cultured overnight in Dulbecco’s Modified Eagle’s medium (DMEM), and the culture was inoculated into fresh DMEM at a ratio of 1:100 for further incubation. When the OD_600_ of the bacterial suspension reached 1.0, the bacterial cells were harvested.

The pellets were resuspended in a lysis buffer, then lysed on ice by ultrasonication for 10 min, and the supernatant was collected. Dithiothreitol (DTT) was added to the supernatant, and the final concentration reached 2 mM. Subsequently, iodoacetamide (IAA) was added to a final concentration of 20 mM. The samples were incubated for 1 h, and proteins were precipitated by adding four volumes of pre-cooled acetone and incubating at −20 °C for at least 2 h. TEAB (pH 8.5) and 8 M urea were added to dissolve the pellet, and protein concentration was determined by Bradford protein assay.

### 2.2. Immunoaffinity Enrichment of Lysine Lactylated Peptides

To digest the treated samples into peptides, trypsin was used at an enzyme-to-protein ratio of 1:20 and incubated at 37 °C for 16 h. The digested peptides were then desalted using a C18 solid-phase extraction cartridge and subsequently dried in a vacuum concentrator. Lyophilized peptides were completely dissolved in MOPS IAP buffer. After centrifugation, the supernatant was incubated with anti-lactyl-lysine beads at 4 °C for 2.5 h. The beads were washed twice with MOPS IAP buffer by centrifugation, then the enriched peptides were eluted from the beads with 0.15% Trifluoroacetic acid (TFA). Finally, the eluate was desalted again using peptide desalting spin columns (Thermo Fisher Scientific, Waltham, MA, USA) prior to LC-MS/MS analysis.

### 2.3. LC-MS/MS Analysis

The mobile phase consisted of solvent A (0.1% formic acid, 2% acetonitrile in water) and solvent B (0.1% formic acid in acetonitrile). Peptide separation was performed on a Bruker Daltonics nanoElute UHPLC system at a constant flow rate 450 nL/min using a linear gradient. Eluted peptides were ionized via electrospray at 1.65 kV in a CaptiveSpray^TM^ nanoBooster source and analyzed on a timsTOF Pro 2 mass spectrometer (Bruker, Billerica, MA, USA). Precursor and fragment ions were detected by the TOF detector, with the mass spectrometer running in data-independent parallel accumulation–serial fragmentation (dia-PASEF) mode. The full MS scan range spanned 100–1700 *m*/*z* (8 PASEF MS/MS scans per cycle), while the MS/MS scan range was 425–1025 *m*/*z* with a 25 *m*/*z* isolation window.

### 2.4. Database Search

Data-Independent Acquisition (DIA) data were database searched using Spectronaut (v17), using the *Vibrio alginolyticus* protein database (containing 4335 sequences) as the reference sequence. The digestion method was set as Trypsin/P, with a maximum of 4 allowable missed cleavage sites. A reverse database was constructed to evaluate the false discovery rate (FDR), and the FDR at the protein, peptide, and PSM levels was controlled within 1%, only proteins supported by at least one unique peptide were accepted as confident identification results. Lysine lactylation sites with localization probability ≥0.75 were considered confidently localized and included in subsequent analyses. The mass spectrometry data have been deposited in Figshare (https://figshare.com/, DOI:10.6084/m9.figshare.31889788).

### 2.5. Cloning the cobQ Gene from Vibrio alginolyticus

The *cobQ* gene was amplified from genomic DNA of *Vibrio alginolyticus* strain HY9901 using gene-specific primers (forward: 5′-ATGATTGTTTGGAGTGTAGCTAAC–3′; reverse: 5′-TTACTGCTCATCGAACCGCAAGTC–3′) and PrimeSTAR^®^ Max DNA Polymerase (TakaraBio, Kusatsu, Japan). PCR conditions included an initial denaturation at 98 °C for 1 min, followed by 30 cycles of 98 °C for 10 s, 60 °C for 30 s, and 72 °C for 1 min, with a final extension at 72 °C for 5 min. The resulting amplicon was purified and directly submitted for Sanger sequencing (Sangon Biotech, Shanghai, China). The obtained sequence was verified by BLAST (DNAman, v9.0) and used for subsequent phylogenetic analysis.

### 2.6. Bioinformatics

The MoMo online software (v5.5.9; https://meme-suite.org/meme/, accessed on 13 July 2025) was used to analyze amino acid sequence motifs. The Clusters of Orthologous Groups of proteins (COG) was analyzed using the NCBI COG database (https://www.ncbi.nlm.nih.gov/COG/, accessed on 25 July 2025). The Gene Ontology (GO) annotation and KEGG pathway were enriched by Omicsbean online software (http://www.omicsbean.cn/). Protein–protein interaction (PPI) networks were predicted by STRING (v12.0; https://string-db.org/) and visualized with Cytoscape (v3.10.0). Phylogenetic analysis was performed with MEGA 5. We predicted the protein domain with SMART (v9.0; https://smart.embl.de/, accessed on 11 January 2026).

### 2.7. CO-IP and Western Blot

To immunoprecipitate target proteins, specific polyclonal antibodies against Crp and Tpi were incubated with cell lysates. Antibodies were subsequently incubated with *V. alginolyticus* cell lysates overnight at 4 °C. Protein A/G beads were washed with PBS and added to the lysates at 4 °C for 2 h, followed by five washes with cold PBS. Then 50 μL of loading sample buffer (250 mM Tris-HCl, pH = 6.8, 10% SDS, 0.5% bromophenol blue, 50% glycerol, and 5% β-mercaptoethanol) was added to the pellet, subsequently analyzed by SDS-PAGE and Western blotting.

Protein samples (10 μg per lane) were loaded onto 10% SDS-PAGE gels and electrophoresed at 200 V for 30 min and electrotransferred onto a polyvinylidene difluoride (PVDF) membrane (Millipore, Burlington, MA, USA). The membrane was blocked with QuickBlock^TM^ Blocking Buffer (Beyotime Biotechnology, Shanghai, China) for 15 min at room temperature. It was then incubated with particular primary antibodies, including anti-Crp, anti-Tpi, and Anti-L-Lactyl-Lysine Rabbit mAb (PTM Biolabs, Hangzhou, China), at a dilution of 1:4000. The membrane was incubated with Horseradish peroxidase (HRP) conjugated goat anti-mouse IgG or anti-rabbit IgG (1:5000) for one hour at room temperature following three washes with TBST (TBS + 0.1% Tween-20) for ten minutes each. Finally, protein bands were visualized using enhanced chemiluminescence (ECL) substrate and captured with a chemiluminescent imaging system.

## 3. Result and Discussion

### 3.1. Identification of Lysine-Lactylated Peptides and Proteins in V. alginolyticus

**Figure 1:**
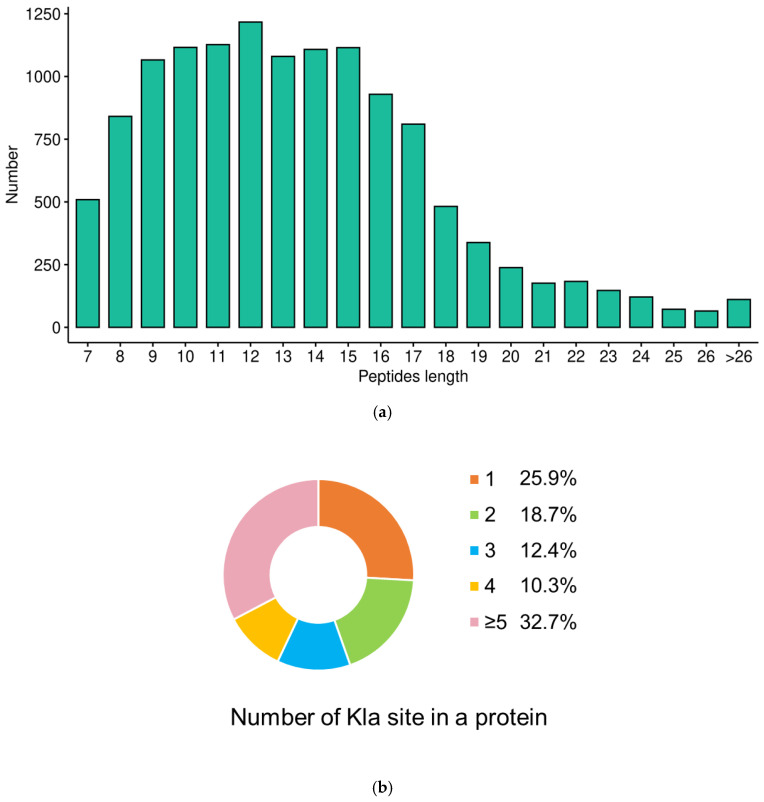
Identification of lysine-lactylated peptides and proteins: (**a**) Length distribution of lactylation-modified peptides in *Vibrio alginolyticus*. (**b**) Analysis of the proportion of lactylation sites in proteins of *Vibrio alginolyticus*.

In this study, the lysine lactylation modification profiles of the bacterial pathogen *V. alginolyticus* were first plotted. Based on the identification results from mass spectrometry analysis and database searching, a total of 9308 Kla sites were identified, corresponding to 9279 modified peptides, and these sites were ultimately annotated to 2155 lactylated proteins (Supplemental Tables S1 and S2). In terms of peptide length distribution, the majority of peptides were distributed in the range of 7–20 amino acids (Figure 1a). This distribution characteristic is consistent with the general rules of enzymatic hydrolysis and mass spectrometry fragmentation methods, which further verifies the reliability of the lactylated identification results in this study. Compared with previous studies on PTMs in *Vibrio alginolyticus*, the modification level of lactylation (49.7%) was higher than that of acetylation (27.1%) and succinylation (15.4%) [23,24], suggesting that lysine lactylation may be a prevalent modification in *Vibrio alginolyticus*, which warrants further investigation into its potential regulatory roles. Additionally, this study analyzed the quantitative distribution of Kla modification sites in proteins. The results revealed that proteins harboring a single Kla site accounted for 25.9%, while those with 2, 3, and 4 Kla sites represented 18.7%, 12.4%, and 10.3% of the total, respectively. Notably, proteins containing 5 or more Kla sites were the most abundant, corresponding to 32.7% (Figure 1b), suggesting that lactylation may regulate protein function through the synergistic effects of multiple modification sites.

### 3.2. Analysis of Lactylated-Lysine Sequence Motifs

**Figure 2:**
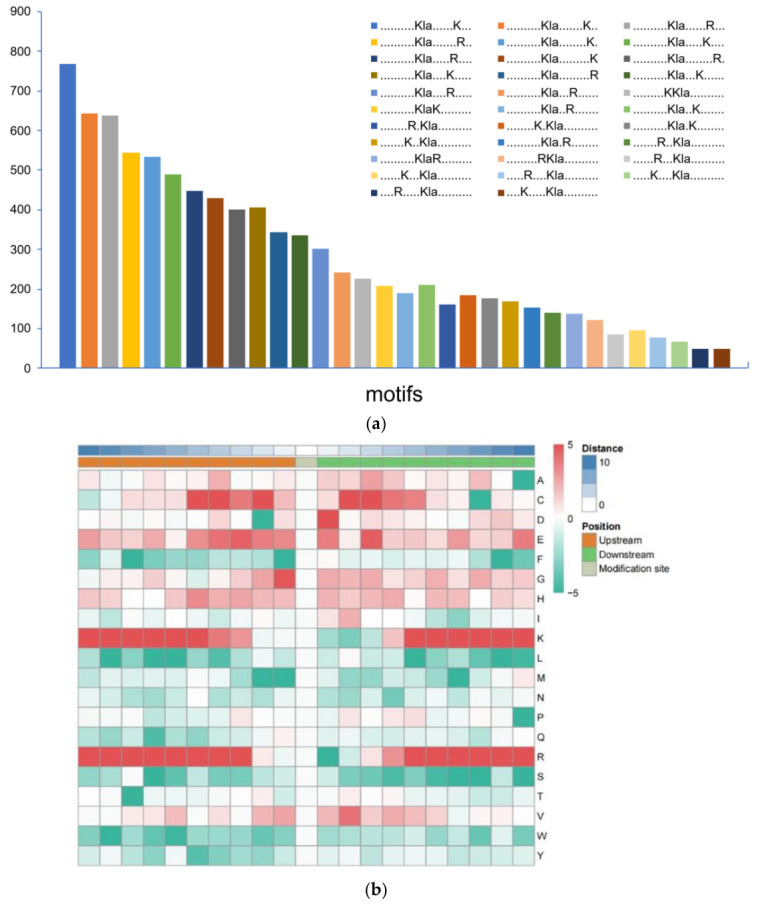
Motif analysis of Kla sites: (**a**) Statistics on the number of distinct motifs for lactylation modification in *Vibrio alginolyticus*. (**b**) Heatmap of amino acid frequency adjacent to lactylation modification sites.

To investigate the preferred recognition sequences of lactylation-modifying enzymes, this study analyzed 10 amino acid sequences flanking each identified Kla site and conducted motif feature analysis through the online tool MoMo (*p* < 0.000001). The results are shown in Figure 2a: a total of 32 conserved motifs were identified near Kla sites, exhibiting distinct abundances. Notably, this number is substantially higher than the number of conserved motifs identified for succinylation (4 motifs) and acetylation (7 motifs), and significantly exceeds the 25 Kla conserved motifs reported in *Candida albicans* [25], indicating that lactylation modification in *Vibrio alginolyticus* exhibits a greater diversity in sequence context preference. As shown in Figure 2b, lysine (K) and arginine (R) were significantly enriched at multiple positions, particularly K in the –10 to –5 and +5 to +10 regions, and R in the −10 to −5 and +4 to +10 regions. The enrichment of these basic residues near lactylation sites suggests they may play a role in defining the preferred sequence context for lactylation in *V. alginolyticus*.

### 3.3. Functional Annotation of Lysine Lactylated Proteins in Vibrio alginolyticus

**Figure 3:**
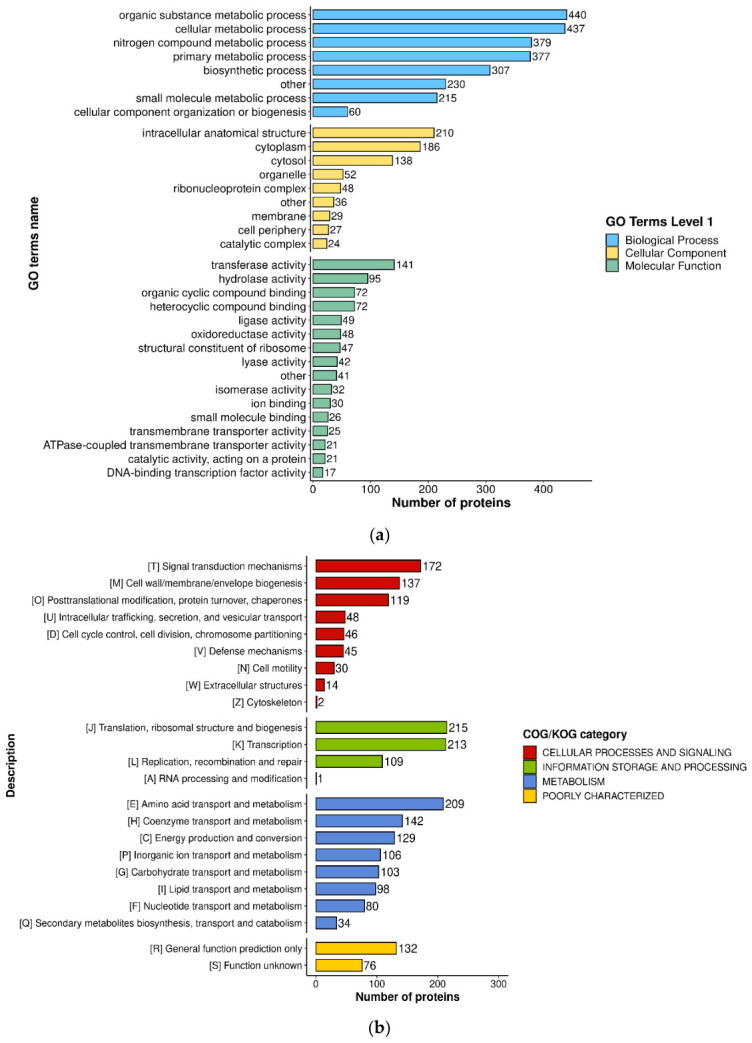

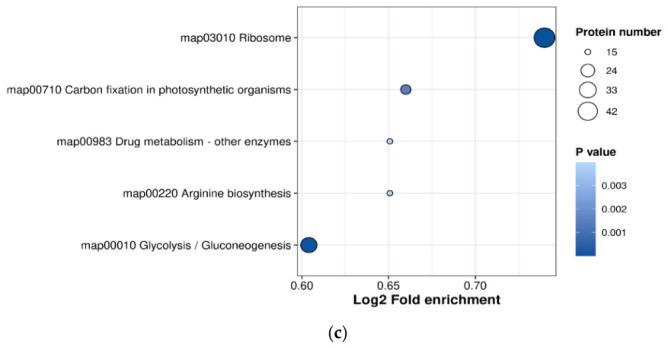
Functional annotation of Kla proteins in *V. alginolyticus*: (**a**) GO enrichment analysis of lactylated proteins. (**b**) The COG function classification analysis. (**c**) The top 5 most significantly enriched functions of the KEGG pathway.

As a post-translational modification directly linked to cellular lactate levels [26], lysine lactylation has been implicated in metabolic regulation in various organisms. A total of 2155 proteins were functionally annotated using multiple databases, including GO, COG, and KEGG databases (Figure 3).

GO annotations were categorized into three main categories: biological processes, cellular components, and molecular functions. In the biological process category, significantly enriched terms were primarily associated with organic substance process, cellular metabolic process, nitrogen compound metabolic process, and primary metabolic process (Figure 3a). Cellular component analysis revealed that the lysine lactylated proteins were mainly related to intracellular anatomical structure, cytoplasm, and cytosol. As for molecular function, transferase activity was the most significantly enriched term, followed by hydrolase activity, organic cyclic compound binding, and heterocyclic compound binding.

To understand the role of lysine lactylated proteins in different biological processes, COG functional annotation was used to analyze the lysine lactylated proteins. As shown in Figure 3b, lysine lactylated proteins were mainly involved in several key Cellular processes and signaling, including signal transduction mechanisms, cell wall/membrane/envelope biogenesis and posttranslational modification, protein turnover, and chaperones. For Information storage and processing, lactylated proteins were enriched in translation, ribosomal structure and biogenesis, transcription, replication, recombination, and repair. Moreover, within the Metabolism category, a significant number of lactylated proteins participated in amino acid transport and metabolism, coenzyme transport and metabolism, energy production and conversion, and others.

The KEGG analysis results indicated that enrichment was achieved in 65 metabolic pathways, with the top 5 enrichment pathways being ribosome, glycolysis/gluconeogenesis, drug metabolism–other enzymes, and arginine biosynthesis (Figure 3c). Among them, lactylated proteins in the ribosome and glycolytic/gluconeogenic metabolic pathways were the most enriched, with 48 and 31 lactylated proteins, respectively. The KEGG enrichment results of the Photobacterium damselae subsp. damselae, which also belongs to the Vibrionaceae family, show that lactylated proteins are highly enriched in the ribosome and glycolysis/gluconeogenesis pathways, and occupy the most significant position in the ribosome pathway [27].

### 3.4. Protein Domain Enrichment Analysis and Subcellular Localization Analysis

**Figure 4:**
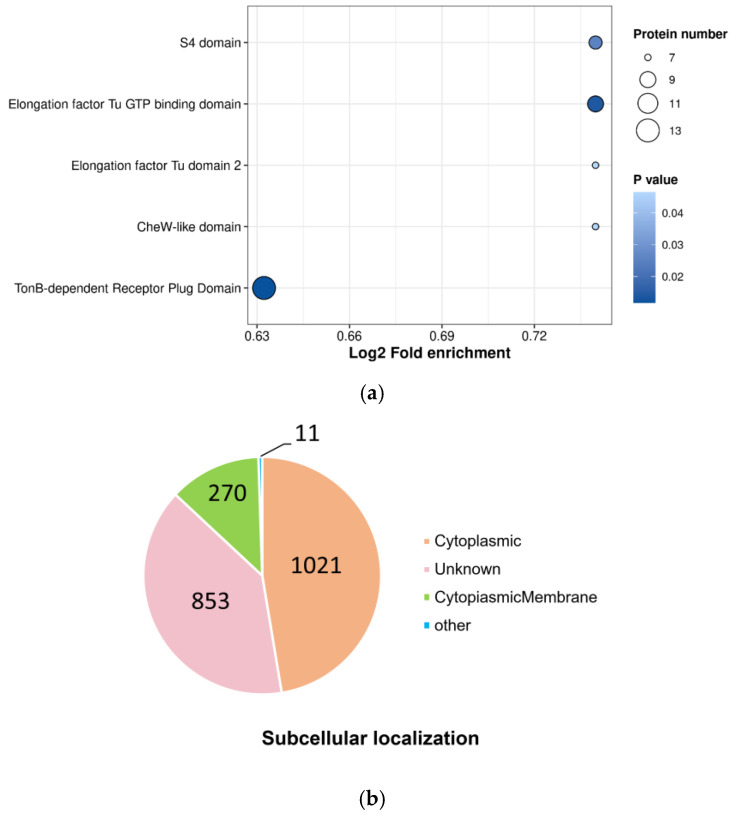
Protein domain enrichment analysis and subcellular localization analysis of lactylation-modified proteins in *V. alginolyticus*: (**a**) protein domain enrichment analysis and (**b**) subcellular localization analysis.

Protein domain enrichment revealed that lysine lactylated proteins had the following domains: TonB-dependent receptor plug domain, elongation factor Tu GTP binding domain, S4 domain, elongation factor Tu domain, and CheW-like domain (Figure 4a). The enrichment of lactylated proteins in the TonB-dependent receptor plug domain suggests a high probability that they are focused on the active iron ion uptake function [28]. Given that iron ions are essential nutrients for bacterial colonization and pathogenicity in the host [29], this implies that these proteins may be involved in processes such as bacterial survival, reproduction, and virulence.

To further explore the functional context of lactylated proteins, we performed subcellular localization analysis, and the results revealed that the majority of the lactylated proteins are localized in the cytoplasm (47.38%), and 270 lactylated proteins were found in the cytoplasmic membrane (12.53%) (Figure 4b). Consistent with the GO cellular component annotation, the cytoplasm is the main location where lactylated proteins perform their functions.

### 3.5. Validation of CRP and TPI Lysine-Lactylated Proteins Using Co-Immunoprecipitation and Western Blotting

**Figure 5:**
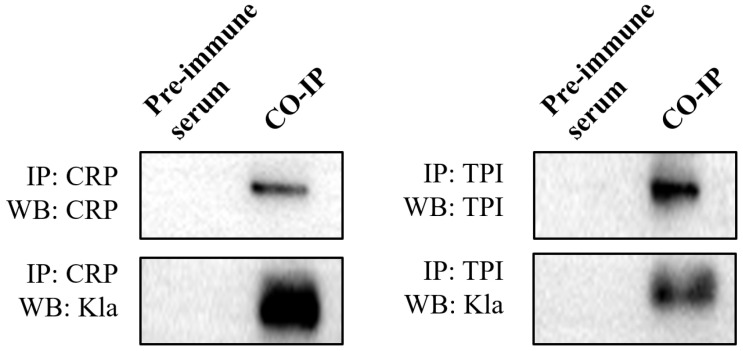
Validation of CRP and TPI lysine lactylation by Co-IP and Western blot. Immunoprecipitated proteins were detected with anti-target (**top**) and anti-Kla (**bottom**) antibodies.

Two candidate proteins, cAMP receptor protein (CRP) and triosephosphate isomerase (TPI), were chosen and examined using Co-IP followed by Western blotting to confirm the lysine lactylated results. Following the capture of the CRP and TPI proteins by their corresponding antibodies, Western blotting was carried out using anti-lactylation and anti-target protein antibodies, respectively. The results demonstrated that CRP and TPI proteins exhibited lactylation modifications consistent with lysine lactylated proteomic data, further supporting our proteomics results (Figure 5).

### 3.6. Overlap Between Lysine Lactylation, Succinylation and Acetylation in V. alginolyticus

Post-translational modifications do not function in isolation. Their overlap on proteins and modification sites forms complex regulatory networks, which collectively regulate the biological functions of proteins [30]. Therefore, we aligned the lysine lactylation results with prior acetylation and succinylation data focusing on PTM proteins and modification sites [25,31].

**Figure 6:**
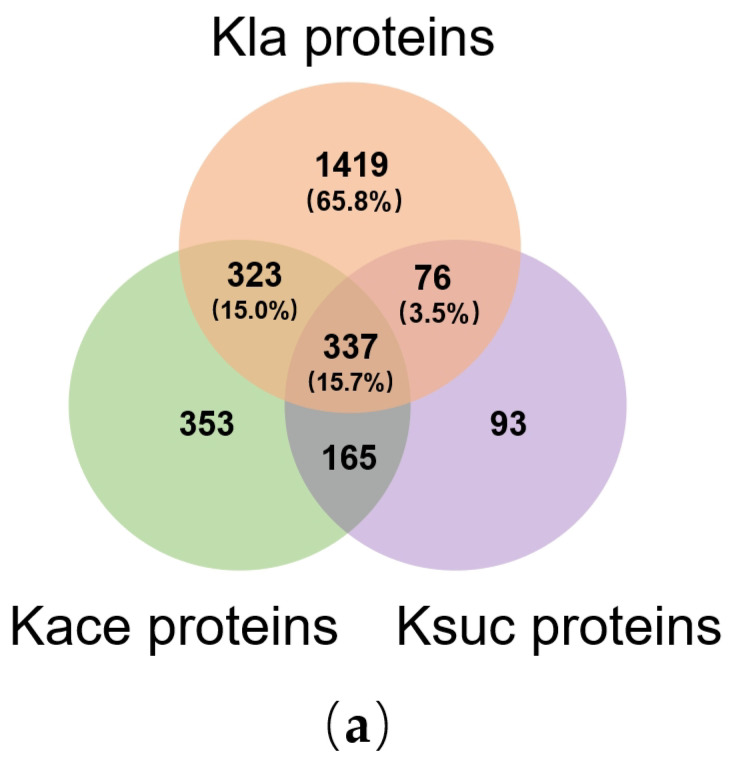

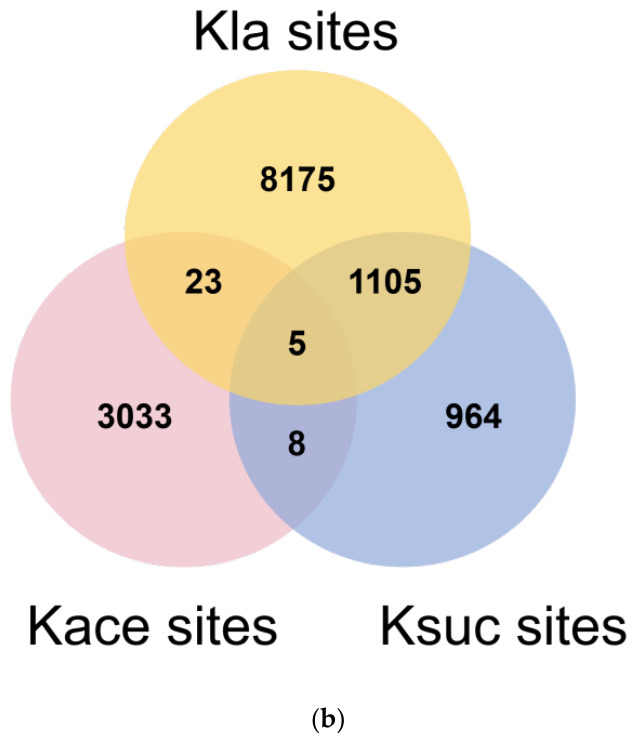
Comparison of three types of PTMs proteins and sites in *V. alginolyticus*: (**a**) Overlap modified proteins in *V. alginolyticus*. Percentages indicate the proportion of each subset relative to the total number of Kla proteins. (**b**) Overlap modified sites in *V. alginolyticus*.

As shown in Figure 6a, a total of 2155 Kla proteins were identified at the protein level. Among them, 65.8% were uniquely identified as lactylated. There was a 15.0% overlap between Kla and Kace proteins, and 3.5% overlap between Kla and Ksuc proteins. Additionally, 337 proteins were modified by all three types of PTM. These results show that Kla proteins account for a relatively high proportion of the three types of modified proteins. Moreover, the overlap degree between Kla and Kace proteins is higher than that between Kla and Ksuc proteins, suggesting that lactylation and acetylation modifications may have a broader functional crosstalk at the protein level.

In contrast, analysis at the lysine residue level (Figure 6b) revealed distinct patterns. A total of 8175 sites were exclusively modified by Kla, with 23 sites overlapping solely with Kace sites and 1105 sites overlapping only with Ksuc sites. Notably, merely 5 sites were commonly shared across all three modifications (Table 1). The overlap at the site level was far lower than that at the protein level, indicating that although the three modifications may target the same protein, they exhibit strong specificity in modifying specific lysine residues. Specifically, the Kla modification shows significant uniqueness at the site level.

### 3.7. Lactylated Proteins of Vibrio alginolyticus in Central Metabolic Pathways

**Figure 7:**
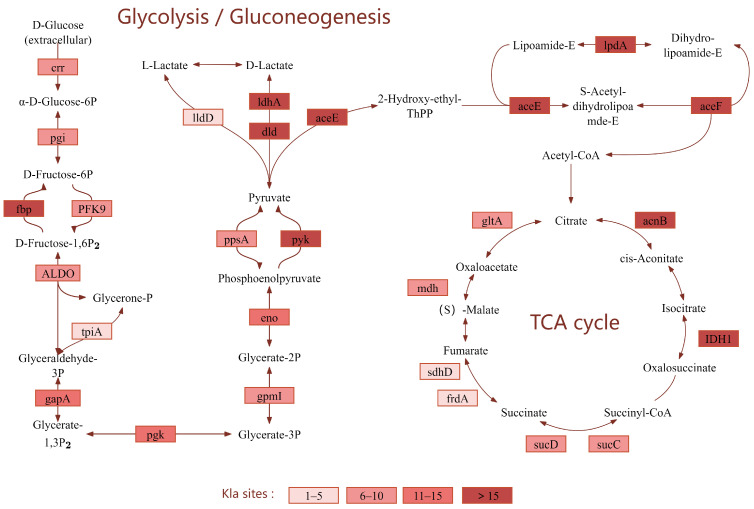
Schematic diagram of key metabolic pathways (Glycolysis/Gluconeogenesis, TCA cycle) involving lactylated proteins.

To explore the functional relevance of lactylation in central metabolism, we focused on two key aspects. First, regarding lactate metabolism, the interconversion between L-lactate and D-lactate—catalyzed by enzymes such as LldD and LdhA (Figure 7)—represents a key step in lactate metabolism. Lactate can serve as a potential precursor of lactyl-CoA during metabolism; however, direct endogenous metabolic evidence is still needed to verify whether lactyl-CoA is the primary acyl donor for bacterial lysine lactylation. Second, we examined the crosstalk between lactylation and major energy-generating pathways. The glycolysis/gluconeogenesis pathway provides energy and material basis for cellular metabolism, while the TCA cycle serves as the core hub of energy metabolism. Lactylation of enzymes or proteins in these pathways can influence coordination between lactate and energy metabolism by regulating enzyme activity, protein–protein interactions, and other mechanisms.

### 3.8. Protein–Protein Interaction Network of Lysine-Lactylated Proteins in Vibrio alginolyticus

**Figure 8:**
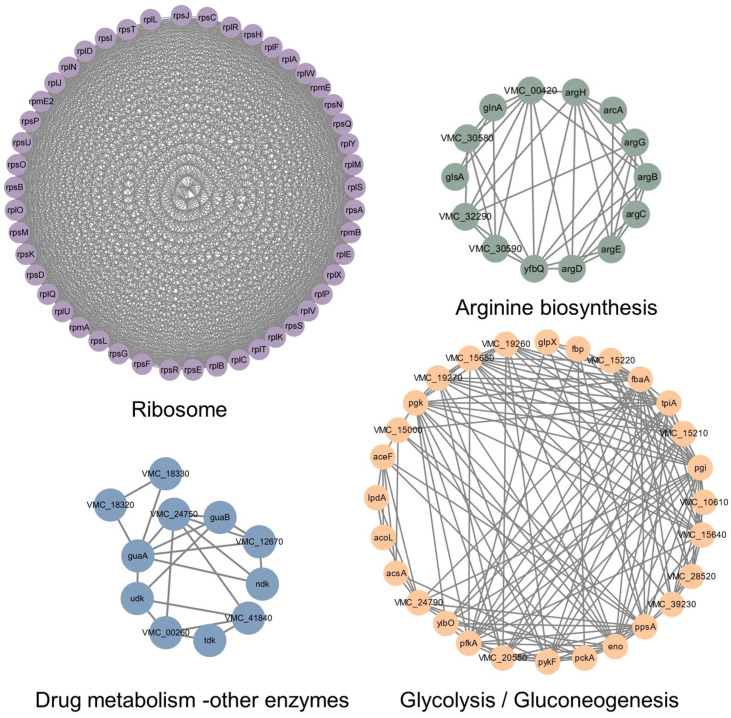
Organization of the *V. alginolyticus* lactylome into functional modules via PPI analysis.

In this study, a protein–protein interaction (PPI) network comprising lysine lactylated proteins was built using the STRING database and Cytoscape 3.10.0 to investigate the possible connections of lactylated proteins. The resulting network exhibits high connectivity (Figure 8), with densely interconnected clusters corresponding to key biological processes. Analysis of the KEGG pathway enrichment and clustering results of the PPI network reveals that there are 4 highly correlated functional modules within the network, which are mainly concentrated in ribosome, glycolysis/gluconeogenesis, arginine biosynthesis, and drug metabolism–other enzymes.

### 3.9. Identification of Kla Sites on T3SS Regulatory Proteins

To identify potential connections between lactylation and known virulence regulators, we compared the previously reported bacterial T3SS regulators with the identified lactylation modification profiles of *Vibrio alginolyticus* proteins, leading to the screening of 8 regulators [31,32,33,34,35]. These proteins cover multiple functional categories, including global transcriptional regulators, a two-component system, an alternative sigma factor, and an sRNA chaperone (Table 2).

The cAMP-activated global transcriptional regulator CRP is modified by lactylation. Previous studies have shown that the cAMP signaling pathway can activate the expression of the major T3SS regulator (ExsA) in Gram-negative bacteria by regulating the activity of global transcription factors, thereby affecting T3SS assembly and effector protein secretion [36]. H-NS is a key transcriptional silencer in Gram-negative bacteria that binds to AT-rich pathogenicity islands harboring T3SS genes, thereby repressing their expression [37]. Activation of T3SS1 requires relief of H-NS-mediated repression followed by induction of *exsA*, the master transcriptional activator of T3SS1, which is directly upregulated by HlyU [38]. According to the study of Liu, acetylation of H-NS at K120 directly reduces its binding affinity to AT-rich DNA, thereby relieving the inhibitory effect on the transcription of the virulence system and significantly enhancing the expression of T3SS [39]. More investigations are necessary to evaluate if the lactylation of H-NS can also diminish its ability to repress transcription, thereby boosting T3SS1 expression.

### 3.10. VaCobQ: A Candidate Delactylase with No Homology to Known Sirtuin Family Deacylases

The aforementioned proteomics analysis not only mapped the lactylation modification profile of *Vibrio alginolyticus* but also revealed an underlying complex regulatory network. To explore the dynamic regulatory mechanism of lactylation modification, we focused on the potential regulatory enzymes. Among the reported deacylation-related proteins, CobB is relatively well characterized and belongs to the sirtuin protein family—a class of nicotinamide adenine dinucleotide (NAD^+^)-dependent class III lysine deacetylases (KDACs) [10,40]. Studies on *Aeromonas hydrophila* have shown that CobQ is a novel deacetylase protein that does not belong to the KDACs [41]. This suggested that CobQ might represent a distinct class of regulatory enzyme. Among the lactylated proteins identified, we also found the presence of CobQ. Therefore, we set out to characterize and validate its potential as a delactylase in *V. alginolyticus*.

**Figure 9:**
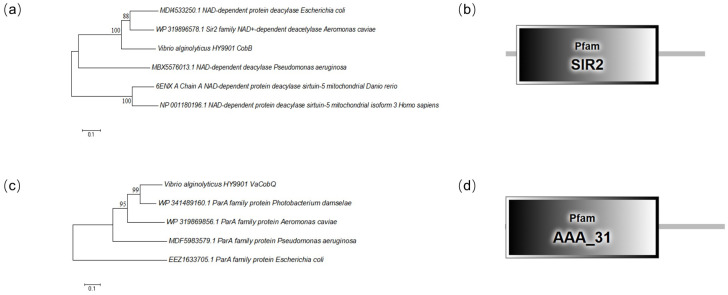
The VaCobB and VaCobQ proteins of *Vibrio alginolyticus* are not homologous: (**a**) The phylogenetic tree of VaCobB. (**b**) The protein domain characteristic of VaCobB. (**c**) The phylogenetic tree of VaCobQ. (**d**) The protein domain characteristic of VaCobQ.

An analysis was conducted to study the characteristics of the CobB and CobQ proteins, which involved comparing the amino acid sequences of VaCobB and VaCobQ from *Vibrio alginolyticus* with their homologous proteins from other species. Figure 9 illustrates the distinct classifications of VaCobB and VaCobQ. VaCobB is grouped within a protein family that includes the deacetylase CobB from *Escherichia coli*, *Pseudomonas aeruginosa*, and *Aeromonas caviae*, in addition to Sirtuin-5 from zebrafish and humans (Figure 9a); all members of this family contain the SIR2 Pfam domain (Figure 9b). Conversely, VaCobQ is homologous to the ParA family proteins of *P. damselae*, *A. caviae*, *P. aeruginosa*, and *E. coli*, and contains the AAA_31 Pfam domain (Figure 9c,d). It was found that these two proteins belong to completely different families of proteins. These results indicate that the CobQ of *Vibrio alginolyticus* shares no homology with the known delactylase CobB.

### 3.11. In Vitro and In Vivo Evidence Suggesting a Role for VaCobQ in Delactylation

**Figure 10:**
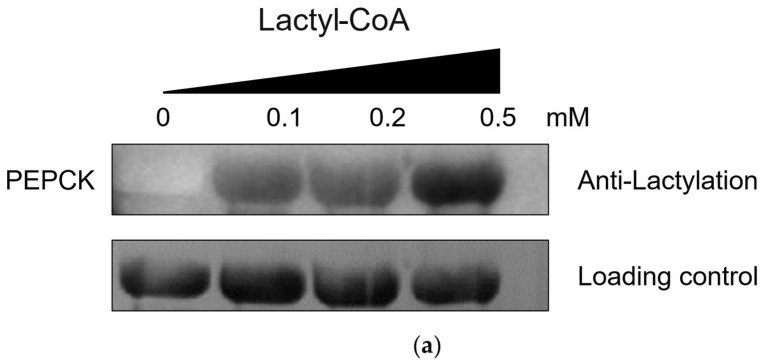

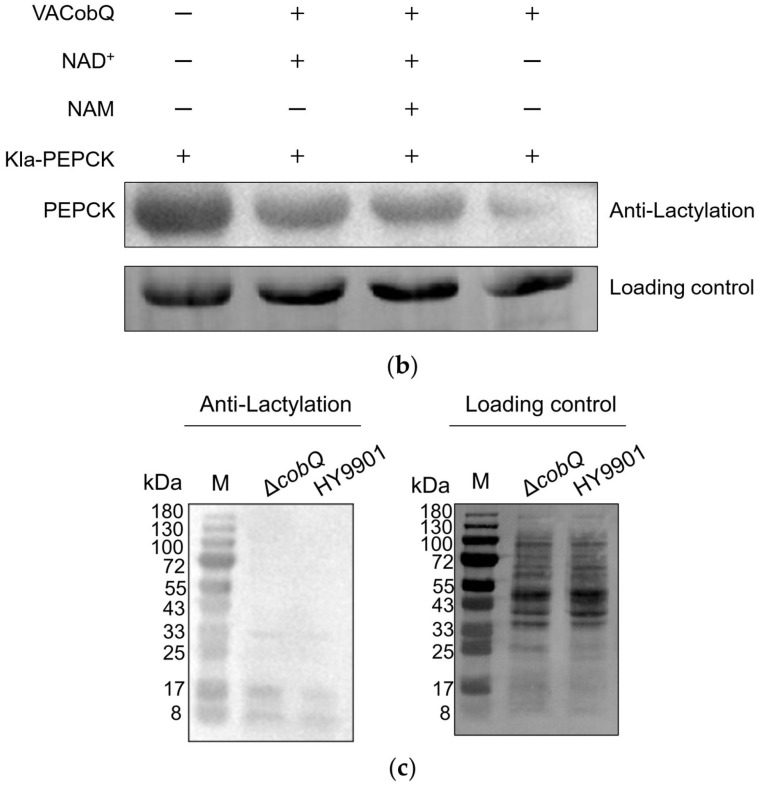
*Vibrio alginolyticus* CobQ is a NAD^+^ independent delactylase: (**a**) In vitro lactylation WB results of PEPCK. (**b**) WB results of lactic acid modification changes in PEPCK after incubation of VaCobQ and lactalyted PEPCK under NAD^+^ and NAM, respectively. (**c**) WB results of global lysine lactylation levels in wild-type, Δ*cobQ* of *Vibrio alginolyticus*.

To validate and characterize the delactylase activity of VaCobQ, assays were performed following the protocol described by Wang [41]. We first performed in vitro lysine lactylation modification on the phosphoenolpyruvate carboxykinase (PEPCK) protein available in our laboratory to obtain lactylated PEPCK (Kla-PEPCK, Figure 10a), then incubated with VaCobQ in a delactylation assay. Results showed that VaCobQ was able to delactylate Kla-PEPCK regardless of the presence or absence of NAD^+^ (Figure 10b). Furthermore, when the NAD^+^-competitive inhibitor NAM was added, the delactylase activity of VaCobQ remained unaffected. To investigate whether VaCobQ modulates protein lactylation in vivo, we constructed a Δ*cobQ* deletion mutant and assessed global Kla levels relative to the wild-type strain HY9901 via anti-Kla Western blotting. The Δ*cobQ* showed a significant elevation in total Kla levels (Figure 10c). On the whole, these results suggest that VaCobQ may function as an NAD^+^-independent delactylase in *V. alginolyticus*. Further exploration including direct binding validation, kinetic characterization, identification of endogenous substrates by proteomics, and assessment of site-specific functional effects will help clarify its regulatory mechanism in future studies.

## Figures and Tables

**Table 1:** Detailed information on the overlapping sites of the three types of PTMs.

Gene Name	Protein Description	Overlap Site
*pur*T	Formate-dependent phosphoribosylglycinamide formyltransferase	[K340]
*pep*A	Probable cytosol aminopeptidase	[K262]
*pnp*	Polyribonucleotide nucleotidyltransferase	[K532]
*suc*B	Dihydrolipoyllysine-residue succinyltransferase component of 2-oxoglutarate dehydrogenase complex	[K153]
*pyr*D	Dihydroorotate dehydrogenase (quinone)	[K207]

**Table 2:** Lactylated proteins associated with the type III secretion system (T3SS) in *Vibrio alginolyticus*.

Protein Name	Protein Description	Kla Sites	Sources
CRP	cAMP-activated global transcriptional regulator	[K5;K23;K27;K36;K53;K90;K101;K153;167;K189;K202]	[32]
PhoB	Two-component sensor	[K91;K105;K110;K204;K213]	[31]
PhoR	Two-component sensor	[K154;K304]	[31]
AphB	Regulator	[K2;K24;K54;K94;K103;K203;K271;K277]	[33]
Hfq	sRNA chaperone protein	[K3]	[34]
H-NS	Regulator	[K6;K57;K99;K108]	[33]
Fur	Ferric uptake regulator protein	[K59;K151]	[31]
RpoN	Regulator	[K2;K265;K298;K310;K312;K339;K343;K354;K400;K438;K447]	[35]

## Data Availability

The raw mass spectrometry data generated in this study have been deposited in Figshare (https://figshare.com/) and are available under DOI: 10.6084/m9.figshare.31889788. The remaining data are contained within the article and Supplementary Materials.

## Supplementary Materials

The following supporting information can be downloaded at: https://www.mdpi.com/article/10.3390/microorganisms14040926/s1, Table S1: The identified lactylated proteins and sites in *V. alginolyticus*; Table S2: The identified lactylated peptides in *V. alginolyticus*.
